# Diastolic dysfunction is associated with an increased risk of contrast-induced nephropathy: a retrospective cohort study

**DOI:** 10.1186/1471-2369-14-146

**Published:** 2013-07-13

**Authors:** Hyang Mo Koo, Fa Mee Doh, Kwang Il Ko, Chan Ho Kim, Mi Jung Lee, Hyung Jung Oh, Seung Hyeok Han, Beom Seok Kim, Tae-Hyun Yoo, Shin-Wook Kang, Kyu Hun Choi

**Affiliations:** 1 Department of Internal Medicine, College of Medicine, Yonsei University, 134 Shinchon-dong, Seodaemun-gu, Seoul, Korea

**Keywords:** Contrast-induced nephropathy, Diastolic dysfunction, E/E’

## Abstract

**Background:**

Contrast-induced nephropathy (CIN) is the third leading cause of hospital-acquired acute kidney injury, and it is associated with poor long-term clinical outcomes. Although systolic heart failure is a well-known risk factor for CIN, no studies have yet evaluated the association between diastolic dysfunction and CIN.

**Methods:**

We conducted a retrospective study of 735 patients who underwent percutaneous transluminal coronary angioplasty (PTCA) and had an echocardiography performed within one month of the procedure at our institute, between January 2009 and December 2010. CIN was defined as an increase of ≥ 0.5 mg/dL or ≥ 25% in serum creatinine level during the 72 hours following PTCA.

**Results:**

CIN occurred in 64 patients (8.7%). Patients with CIN were older, had more comorbidities, and had an intra-aortic balloon pump (IABP) placed more frequently during PTCA than patients without CIN. They showed greater high-sensitivity C-reactive protein (hs-CRP) levels and lower estimated glomerular filtration rates (eGFR). Echocardiographic findings revealed lower ejection fraction and higher left atrial volume index and E/E’ in the CIN group compared with non-CIN group. When patients were classified into 3 groups according to the E/E’ values of 8 and 15, CIN occurred in 42 (21.6%) patients in the highest tertile compared with 20 (4.0%) in the middle and 2 (4.3%) in the lowest tertile (p < 0.001). In multivariate logistic regression analysis, E/E’ > 15 was identified as an independent risk factor for the development of CIN after adjustment for age, diabetes, dose of contrast media, IABP use, eGFR, hs-CRP, and echocardiographic parameters [odds ratio (OR) 2.579, 95% confidence interval (CI) 1.082-5.964, p = 0.035]. In addition, the area under the receiver operating characteristic curve of E/E’ was 0.751 (95% CI 0.684-0.819, p < 0.001), which was comparable to that of ejection fraction and left atrial volume index (0.739 and 0.656, respectively, p < 0.001).

**Conclusions:**

This study demonstrated that, among echocardiographic variables, E/E' was an independent predictor of CIN. This in turn suggests that diastolic dysfunction may be a useful parameter in CIN risk stratification.

## Background

Contrast-induced nephropathy (CIN) is one of the principal complications which develop after procedures using contrast media (CM). Although there are some differences between institutions, the most commonly used definition of CIN in clinical trials is a rise in serum creatinine of > 0.5 mg/dL or > 25% from the baseline value within 72 hours after exposure to the CM. The incidence of CIN is reported to be about 2% in the general population, 7-15% in patients undergoing percutaneous coronary intervention, whereas it increases up to 50% in high-risk patients with diabetes (DM) and renal failure [1,2]. Recently, risk stratification and application of preventive methods against CIN further reduced the incidence of CIN. However, CIN is still an important problem in that dependence for medical procedures using CM is steadily increasing and that it is closely related with poor long-term clinical outcomes: longer hospital stay, and increased mortality and post-procedural cardiovascular complications [3,4].

Accordingly, many investigators have tried to reveal the pathogenesis of CIN. Delineated mechanisms include intra-renal vasoconstriction, reduced renal blood flow, medullary hypoxia, oxidative stress, inflammation, endothelial dysfunction, and direct tubular-epithelial cell injury by CM [5]. At the same time, risk factors potentiating the processes explained above were proved out: renal failure, old age, DM, congestive heart failure (CHF), hypotensive event, use of intra-aortic balloon pump (IABP), and ionic/ high-osmolar CM [6].

CHF, especially in advanced stages of New York Heart Association (NYHA) class 3–4 contributes to the development of CIN primarily by decreasing renal perfusion. It is related with systolic dysfunction and low stroke volume [7]. In fact, some researches specifically pointed ejection fraction (EF) < 30-40% out as an independent predictor of CIN [8]. However, CHF per se and just a past history of heart failure increased the incidence of CIN, irrespective of EF [9,10]. Nevertheless, no studies have yet evaluated the association between diastolic dysfunction and CIN.

The golden standard estimating diastolic function of the heart is to measure left ventricular end-diastolic pressure (LVEDP) with catheterization. However, it is invasive and not a routine practice. On the other hand, E/E’ can be assessed non-invasively with echocardiography, and is known to be less influenced by heart rate, atrial activity, or ejection fraction [11]. In addition, E/E’ levels were well correlated with LVEDP, when patients were categorized into 3 groups based on the E/E’ values of 8 and 15 [12]. E/E’ > 11 also predicted LVEDP > 15 mmHg with a sensitivity of 75% and a specificity of 93% [13]. Among various doppler estimates of diastolic function such as E, E/A, decrease in E/A with the valsalva maneuver, deceleration time (DT), and pulmonary venous atrial reversal duration, E/E’ showed the highest predictive power for LVEDP [12].

So, we aimed to investigate whether E/E’ is an independent risk factor predicting the development of CIN. Effects of E/E’ and CIN on patient mortality were also evaluated.

## Methods

### Ethics statement

This study was carried out in accordance with the Declaration of Helsinki and approved by the Institutional Review Board (IRB) of Yonsei University Health System Clinical Trial Center. Written consents were not required because this was a retrospective medical record-based study and personally identifiable information was anonymized.

### Patients

This retrospective study included 735 patients who underwent percutaneous transluminal coronary angioplasty (PTCA) at Severance Hospital in Seoul, Korea from January 2009 to December 2010. PTCA was conducted in 6837 patients for the following reasons: 1) regular follow-up for known coronary artery occlusive disease (CAOD), 2) suspicion for CAOD based on clinical symptoms or study results (treadmill test, technetium sestamibi scan, or cardiac CT or MRI), or 3) to evaluate whether idiopathic CHF was due to ischemia. Among these, 3302 subjects who had undergone echocardiography within 1 month of PTCA were included. Exposure to other CM within 7 days of PTCA (n = 1576), an absence of data on serum creatinine during the 72 hours following the procedure (n = 503), end-stage renal disease (n = 408), underlying malignancy (n = 43), acute infection (n = 28), or age less than 18 years (n = 9) resulted in the exclusion of an additional 2567 patients.

All patients were hydrated with 0.9% saline at a rate of 1.0 mL/kg/hour for 12 hours pre- and post-exposure to the CM, according to the guidelines of our clinic. In case of an emergency procedure, hydration was initiated immediately prior to the start of angiography and continued for 24 hours after PTCA. Infusion rate was reduced to 0.5 mL/kg/hour when pulmonary edema was noted or ejection fraction was less than 30%.

Non-ionic, dimeric, and iso-osmolar contrast agent (iodixanol: Visipaque, GE Healthcare, Amersham, United Kingdom) was used for PTCA. A hypotensive event was defined as systolic blood pressure < 80 mmHg for at least 1 hour requiring inotropic therapy or IABP insertion within 24 hours of procedure, as described by Mehran et al. [14].

### Data collection

Demographic and clinical data at the time of the PTCA were collected through medical chart review. Laboratory findings which were reported 24 hours prior to the PTCA and measured in overnight fasting status, were set as baseline values. CIN was defined as an increase of ≥ 0.5 mg/dL or ≥ 25% in serum creatinine level during the 72 hours following PTCA, that could not be better explained by alternative etiologies. We also used a second definition of CIN according to the Acute Kidney Injury Network (AKIN) criteria: a rise in serum creatinine ≥ 0.3 mg/dL within 48 hours of procedure [3,15]. Oliguria was not considered because all patients were hydrated and some of them used diuretics.

Hemoglobin, albumin, blood urea nitrogen, creatinine, serum cholesterol, triglycerides, and glucose levels were measured by an Advia 2120 Hematology Analyzer (Siemens Health-care Diagnostics, Deerfield, Illinois). High-sensitivity C-reactive protein (hs-CRP) levels were measured with a BN II analyzer (Dade Behring, Newark, DE, USA) using the latex-enhanced immunonephelometric method. The estimated glomerular filtration rate (eGFR) was calculated using the Modification of Diet in Renal Disease (MDRD) equation [16].

### Cardiac status and echocardiographic parameters

Records of PTCA were reviewed for the urgency of the procedure, amount of CM used, interventional strategy, and the number of coronary arteries involved. We also investigated whether any patients had undergone coronary artery bypass graft surgery previously.

Echocardiography was performed with a SONOS 7500 (Philips Ultrasound, Bothell, WA, USA) according to the recommendations of the American Society of Echocardiography (ASE). Inter-ventricular septal thickness (IVSs, IVSd), posterior wall thickness (PWTs, PWTd), left ventricular end-diastolic dimension (LVDd), and left ventricular end-systolic dimension (LVDs) were measured in 2-dimensional M-mode. Pulsed wave doppler was applied to check velocities in the 4-chamber apical view. Volume sampling was positioned at the tip of the mitral valve to measure early LV filling velocity (E) and left atrial contraction velocity (A). Tissue doppler was then conducted with the volume sampling repositioning at the septal annulus of the mitral valve to measure early (E’) and late (A’) diastolic mitral annular velocities.

LV systolic function was defined by EF. The EF was calculated by the modified Simpson’s method, subtracting LVDs from LVDd. LV mass (LVM) was estimated using the Devereux modified ASE cube formula [17], and the LV mass index (LVMI) was calculated by dividing LVM by body surface area.

$$\begin{array}{ll} \mathrm{LVM}\;=\;0.8\; & \;\times \;[1.04\;\times \;(\mathrm{IVSd}\;+\;\mathrm{LVDd}\;\\ & \;+\;\mathrm{PWTd})3-\;\mathrm{LVDd}3]+\;0.6\;(\mathrm{gm}) \end{array}$$

E/E’ and E/A were calculated to represent diastolic function. DT, the time between peak E wave and the upper deceleration slope extrapolated to zero line, was also determined. In 23 patients exhibiting atrial fibrillation (n = 21, 3.1% in non-CIN group and n = 2, 3.1% in CIN group), average measurements from 10 cardiac cycles were used [11].

Left atrial volume index (LAVI), a marker of volume status, was estimated with the biplane Simpson’s method using the diameters of the LA, which were measured 3 times at the parasternal long axis view (anterior-posterior, superior-inferior) and the 4 chamber view (medio-lateral).

Inter-reader reliability, intra-reader reliability, and reader drift analyses were performed on a random sample of 3% of the entire cohort. The intra-class correlation coefficients for the echocardiographic measures were 0.845 for EF, 0.765 for LVMI, and 0.753 for LAVI.

### Statistical analysis

Statistical analysis was performed with the Statistical Package for the Social Sciences (SPSS) for Windows, version 18.0 (Chicago, IL, USA). Data are expressed as a mean ± standard deviation for continuous variables, and as a number and percentage for categorical variables. Normality of distribution was examined by the Shapiro-Wilk test.

We compared the demographic, laboratory, and echocardiographic parameters of patients with CIN with those of patients without CIN using the Student’s t-test, Mann–Whitney U test, or chi-square test. Either ANOVA or the Kruskal-Wallis test was performed to compare the 3 groups, which were classified according to the E/E’ values of 8 and 15. Univariate and multivariate binary logistic regression analyses were conducted to identify risk factors predicting the development of CIN. We also performed receiver operating characteristic (ROC) analysis to confirm the predictive accuracy of echocardiographic parameters for CIN. All-cause mortality was compared between patients using 4 groupings that were based on the presence or absence of diastolic dysfunction (E/E’ > 15) and CIN, by Kaplan-Meier and log-rank test. A p-value of less than 0.05 was considered statistically significant.

## Results

### Baseline characteristics between patients with CIN and those without CIN

Baseline characteristics of the study subjects are detailed in Table 1. The mean age was 64.8 ± 10.6 years and 69.9% were male. The mean values of eGFR and E/E’ were 74.5 ± 20.5 mL/min/1.73m^2^ and 13.3 ± 5.3, respectively. A total of 64 patients (8.7%) developed CIN.

**Table 1:** Baseline characteristics of study subjects

**Variables**	**All (n = 735)**	**CIN (n = 64)**	**No CIN**	**p**
			**(n = 671)**	
Age (years)	64.8 ± 10.6	70.8 ± 10.5	64.2 ± 10.5	<0.001
Sex (male)	514 (69.9%)	39 (60.9%)	475 (70.8%)	0.101
BMI (kg/m^2^)	24.5 ± 3.3	22.7 ± 3.3	24.7 ± 3.3	<0.001
Systolic BP (mmHg)	119.6 ± 15.1	119.5 ± 21.6	119.6 ± 14.4	0.979
Diastolic BP (mmHg)	71.9 ± 9.9	69.0 ± 14.2	72.2 ± 9.3	0.119
Hypertension	475 (64.6%)	52 (81.3%)	423 (63.0%)	0.004
Diabetes mellitus	250 (34.0%)	39 (60.9%)	211 (31.4%)	<0.001
Previous CABG	23 (3.1%)	5 (7.8%)	18 (2.7%)	0.042
Emergency/ urgent procedure	160 (21.8%)	23 (35.9%)	137 (20.4%)	0.004
Interventional strategy				0.521
Ballooning	23 (3.1%)	1 (1.6%)	22 (3.3%)	
Ballooning + Stent insertion	614 (83.5%)	52 (81.3%)	562 (83.8%)	
Ballooning + Stent insertion + Thrombus suction	98 (13.3%)	11 (17.2%)	87 (13.0%)	
3-vessel disease	241 (32.8%)	39 (60.9%)	202 (30.1%)	<0.001
Hypotensive event	90 (12.2%)	25 (39.1%)	65 (9.7%)	<0.001
IABP use	32 (4.4%)	16 (25.0%)	16 (2.4%)	<0.001
Total volume of CM (mL)	237.0 ± 74.5	222.8 ± 71.4	238.1 ± 74.7	0.179
Volume of CM per weight (mL/kg)	3.69 ± 1.31	4.09 ± 1.86	3.66 ± 1.25	0.179
N-acetylcysteine	233 (33.5%)	15 (42.9%)	218 (33.0%)	0.230
BUN (mg/dL)	18.0 ± 7.3	24.2 ± 11.2	17.4 ± 6.6	<0.001
Creatinine (mg/dL)	1.08 ± 0.41	1.50 ± 0.75	1.04 ± 0.33	<0.001
eGFR (mL/min/1.73m^2^)	74.5 ± 20.5	54.5 ± 25.8	76.4 ± 18.9	<0.001
Hemoglobin (g/dL)	13.2 ± 1.8	11.5 ± 2.1	13.3 ± 1.7	<0.001
Albumin (g/dL)	4.31 ± 0.48	3.85 ± 0.61	4.35 ± 0.44	<0.001
hs-CRP (mg/L)	10.18 ± 28.84	34.11 ± 47.95	7.84 ± 24.77	<0.001
Cholesterol (mg/dL)	164.5 ± 43.5	167.5 ± 56.5	164.3 ± 42.2	0.722
Triglyceride (mg/dL)	140.0 ± 82.3	126.0 ± 87.7	141.3 ± 81.7	0.035
Glucose (mg/dL)	124.6 ± 46.3	156.2 ± 71.3	121.6 ± 42.1	<0.001
Ejection fraction (%)	57.5 ± 13.4	48.8 ± 12.8	58.6 ± 12.7	<0.001
E/E'	13.3 ± 5.3	18.2 ± 7.0	12.9 ± 4.9	<0.001
E/A	0.94 ± 0.48	0.95 ± 0.37	0.94 ± 0.49	0.630
Deceleration time (ms)	204.6 ± 50.1	182.5 ± 50.5	206.7 ± 49.7	<0.001
LV mass (g)	213.6 ± 95.7	233.1 ± 56.2	211.9 ± 98.4	0.001
LV mass index (g/m^2^)	126.3 ± 88.6	144.0 ± 28.9	124.7 ± 91.9	<0.001
LVDd (mm)	50.0 ± 5.0	51.2 ± 5.6	49.9 ± 4.9	0.030
LA volume index (mL/m^2^)	28.3 ± 9.7	33.4 ± 11.6	27.8 ± 9.4	<0.001

Abbreviations: *BMI* body mass index; *BP* blood pressure; *BUN* blood urea nitrogen; *CABG* coronary artery bypass graft; *CIN* contrast-induced nephropathy; *CM* contrast media; *eGFR* estimated glomerular filtration rate; *hs*-*CRP* high sensitivity C-reactive protein; *IABP* intra-aortic balloon pump; *LA* left atrium; *LV* left ventricle; *LVDd* left ventricular end-diastolic dimension.

When patients were dichotomized into CIN and non-CIN groups, the CIN group was older and had lower body mass index (BMI) than the non-CIN group. The prevalence of hypertension, DM, emergency/ urgent procedure, three-vessel coronary artery disease (3-VD), previous coronary artery bypass graft surgery, and IABP use during the PTCA was significantly higher in the CIN group compared to the non-CIN group. Within the laboratory findings, patients with CIN demonstrated eGFR, hemoglobin, and albumin levels that were significantly lower, while hs-CRP levels were significantly higher compared to the non-CIN group. Echocardiographic findings revealed that the EF and DT were decreased, while E/E’ (18.2 ± 7.0 vs. 12.9 ± 4.9, p < 0.001), LVMI, LVDd, and LAVI were significantly increased in patients with CIN.

When patients were classified into three groups based on the E/E’ values of 8 and 15, CIN occurred in 42 (21.6%) patients in the highest tertile compared with 20 (4.0%) in the middle and 2 (4.3%) in the lowest tertile (p < 0.001). In addition, patients in the highest tertile were older and had more hypertension, DM, and 3-VD. They also experienced more hypotensive events and had IABPs placed more frequently during procedure than patients in the middle and lowest tertiles. Among the echocardiographic parameters, LVMI, LVDd, and LAVI were significantly increased in the highest tertile (Table 2).

**Table 2:** **Differences in variables according to tertiles of E**/**E**’

**Variables**	**E/E**’ ≤ **8**	**8 <E**/**E**’ ≤ **15**	**E/E’ >15**	*p*
	**(n = 47)**	**(n = 494)**	**(n = 194)**	
Age (years)	57.0 ± 9.5	63.4 ± 10.3	70.1 ± 9.6	0.036
Sex (male)	42 (89.4%)	375 (75.9%)	97 (50.0%)	<0.001
BMI (kg/m^2^)	23.7 ± 2.3	24.7 ± 3.3	24.4 ± 3.6	0.061
Systolic BP (mmHg)	115.3 ± 13.6	119.9 ± 14.0	119.9 ± 17.9	0.018
Diastolic BP (mmHg)	71.3 ± 9.4	72.6 ± 9.4	70.4 ± 10.8	0.150
Hypertension	21 (44.7%)	305 (61.7%)	149 (76.8%)	<0.001
Diabetes mellitus	12 (25.5%)	154 (31.2%)	84 (43.3%)	0.005
Previous CABG	0 (0.0%)	13 (2.6%)	10 (5.2%)	0.130
Emergency/ urgent procedure	5 (10.6%)	103 (20.9%)	52 (26.8%)	0.038
Interventional strategy				0.680
Ballooning	1 (2.1%)	14 (2.8%)	8 (4.1%)	
Ballooning + Stent insertion	42 (89.4%)	415 (84.0%)	157 (80.9%)	
Ballooning + Stent insertion + Thrombus suction	4 (8.5%)	65 (13.2%)	29 (14.9%)	
3-vessel disease	12 (25.5%)	134 (27.1%)	95 (49.0%)	<0.001
Hypotensive event	3 (6.4%)	45 (9.1%)	42 (21.6%)	<0.001
IABP use	0 (0.0%)	11 (2.2%)	21 (10.8%)	<0.001
Total volume of CM (mL)	236.6 ± 89.4	237.9 ± 75.0	234.6 ± 69.1	0.956
Volume of CM per weight (mL/kg)	3.53 ± 1.19	3.62 ± 1.22	3.92 ± 1.53	0.176
N-acetylcysteine	24 (25.3%)	144 (33.2%)	65 (39.2%)	0.071
CIN	2 (4.3%)	20 (4.0%)	42 (21.6%)	<0.001
BUN (mg/dL)	15.6 ± 3.5	17.3 ± 6.3	20.2 ± 9.6	0.112
Creatinine (mg/dL)	1.02 ± 0.25	1.04 ± 0.32	1.21 ± 0.57	0.848
eGFR (mL/min/1.73m^2^)	82.9 ± 17.9	77.4 ± 18.7	64.9 ± 22.4	0.071
Hemoglobin (g/dL)	13.9 ± 1.3	13.4 ± 1.7	12.3 ± 1.9	0.058
Albumin (g/dL)	4.45 ± 0.43	4.39 ± 0.41	4.06 ± 0.54	0.308
hs-CRP (mg/L)	6.85 ± 29.80	7.67 ± 24.10	17.20 ± 36.50	<0.001
Cholesterol (mg/dL)	161.7 ± 42.7	164.6 ± 42.9	165.4 ± 45.7	0.570
Triglyceride (mg/dL)	143.9 ± 95.3	142.5 ± 85.1	132.4 ± 70.4	0.742
Glucose (mg/dL)	116.6 ± 41.3	121.8 ± 41.6	133.7 ± 56.6	0.205
Ejection fraction (%)	61.0 ± 10.0	59.4 ± 12.1	52.7 ± 14.4	0.578
E/E'	6.7 ± 0.8	11.2 ± 2.1	20.4 ± 4.9	<0.001
E/A	0.98 ± 0.38	0.90 ± 0.50	1.01 ± 0.44	0.065
Deceleration time (ms)	207.5 ± 46.1	207.9 ± 49.5	195.6 ± 52.0	0.699
LV mass (g)	188.6 ± 41.6	212.1 ± 108.2	223.6 ± 64.5	0.014
LV mass index (g/m^2^)	107.2 ± 22.6	121.5 ± 57.0	143.5 ± 145.8	0.003
LVDd (mm)	48.4 ± 3.5	49.8 ± 4.7	50.8 ± 5.8	0.014
LA volume index (mL/m^2^)	22.3 ± 4.8	26.6 ± 7.9	34.1 ± 11.9	<0.001

Abbreviations: *BMI* body mass index; *BP* blood pressure; *BUN* blood urea nitrogen; *CABG* coronary artery bypass graft; *CIN* contrast-induced nephropathy; *CM* contrast media; *eGFR* estimated glomerular filtration rate; *hs*-*CRP* high sensitivity C-reactive protein; *IABP* intra-aortic balloon pump; *LA* left atrium; *LV* left ventricle; *LVDd* left ventricular end-diastolic dimension.

### Risk factors for the development of CIN

Logistic regression analysis showed that higher E/E’ level [odds ratio (OR) 1.147, 95% confidence interval (CI) 1.101-1.194, p < 0.001 as a continuous variable/ OR 6.519, 95% CI 3.774-11.260, p < 0.001 as a categorical variable] was a significant risk factor for CIN (Table 3). After adjustment for age, BMI, hypertension, DM, emergency/ urgent procedure, 3-VD, volume of CM per weight, use of IABP, eGFR < 60 mL/min/1.73m^2^, hemoglobin, albumin, and hs-CRP, E/E’ still remained as an independent risk factor (OR 1.091, 95% CI 1.026-1.159, p = 0.005 as a continuous variable/ OR 3.435, 95% CI 1.522-7.755, p = 0.003 as a categorical variable). In successive models, further adjustments were made with other echocardiographic parameters, EF and LAVI. E/E’ > 15 was found to be a final determinant of CIN (OR 2.579, 95% CI 1.082-5.964, p = 0.035) (Table 4).

**Table 3:** **Univariate logistic regression analysis for contrast**-**induced nephropathy**

**Variables**	**OR**	**95% CI**	***p***
Age > 75 years (vs. ≤ 75 years)	2.837	1.038-1.097	<0.001
BMI (kg/m^2^)	0.808	0.738-0.886	<0.001
Hypertension	2.541	1.330-4.852	0.005
Diabetes mellitus	3.401	2.006-55.766	<0.001
Emergency/ urgent procedure	2.187	1.269-3.767	0.005
(vs. elective procedure)			
3-vessel disease (vs. < 3-vessel involvement)	3.622	2.135-6.145	<0.001
IABP use (vs. non-IABP use)	16.872	7.670-37.113	<0.001
Volume of CM per weight (mL/kg)	1.250	1.024-1.526	0.028
eGFR < 60 mL/min/1.73m^2^	11.343	6.434-19.997	<0.001
(vs. eGFR ≥ 60 mL/min/1.73m^2^)			
Hemoglobin (g/dL)	0.571	0.490-0.664	<0.001
Albumin (g/dL)	0.179	0.111-0.289	<0.001
hs-CRP (mg/L)	1.017	1.010-1.023	<0.001
E/E'	1.147	1.101-1.194	<0.001
E/E' > 15 (vs. E/E’ ≤ 15)	6.519	3.774-11.260	<0.001
EF ≤ 40% (vs. EF > 40%)	5.142	2.907-9.096	<0.001
LAVI > 35 mL/m^2^ (vs. LAVI ≤ 35 mL/m^2^)	3.865	2.230-6.700	<0.001

Data are presented as odds ratios (OR) and 95% confidence intervals (CI).

Abbreviations: *BMI* body mass index; *IABP* intra-aortic balloon pump; *CM* contrast media; *eGFR* estimated glomerular filtration rate; *hs*-*CRP*, high sensitivity C-reactive protein; *EF* ejection fraction; *LAVI* left atrial volume index.

**Table 4:** **Odds ratios and 95**% **confidence intervals for contrast**-**induced nephropathy according to the E**/**E**’ **levels** (**Multivariate logistic regression analysis**)

	**E/E**’		
	**OR**	**95% CI**	***p***
^a^Model 1	1.091	1.026-1.159	0.005
^b^Model 2	3.435	1.522-7.755	0.003
^c^Model 3	3.344	1.456-7.682	0.004
^d^Model 4	2.579	1.082-5.964	0.035

^a^ Model 1 (odds ratio per 1 increase in E/E’) : adjusted for age (> 75 years vs. ≤ 75 years), BMI, hypertension, diabetes, emergent/ urgent procedure, 3-vessel disease, IABP use, volume of CM per weight, eGFR (< 60 mL/min/1.73m^2^ vs. eGFR ≥ 60 mL/min/1.73m^2^), hemoglobin, albumin, and hs-CRP.

^b^ Model 2 (odds ratio for E/E’ > 15 vs. E/E’ ≤ 15) : adjusted for age (> 75 years vs. ≤ 75 years), BMI, hypertension, diabetes, emergent/ urgent procedure, 3-vessel disease, IABP use, volume of CM per weight, eGFR (< 60 mL/min/1.73m^2^ vs. eGFR ≥ 60 mL/min/1.73m^2^), hemoglobin, albumin, and hs-CRP.

^c^ Model 3 (odds ratio for E/E’ > 15 vs. E/E’ ≤ 15) : adjusted for Model 2 plus ejection fraction (≤ 40% vs. > 40%).

^d^ Model 4 (odds ratio for E/E’ > 15 vs. E/E’ ≤ 15) : adjusted for Model 3 plus left atrial volume index (> 35 vs. ≤ 35).

Abbreviations: *BMI* body mass index; *IABP* intra-aortic balloon pump; *CM* contrast media; *eGFR* estimated glomerular filtration rate; *hs*-*CRP* high sensitivity C-reactive protein.

Defining CIN as an increase in serum creatinine ≥ 0.3 mg/dL from the baseline value, according to the AKIN criteria, did not change the independent role of E/E’ for the prediction of CIN (OR 2.456, 95% CI 1.046-6.217, p = 0.044) (see Additional file 1).

**Figure 1:**
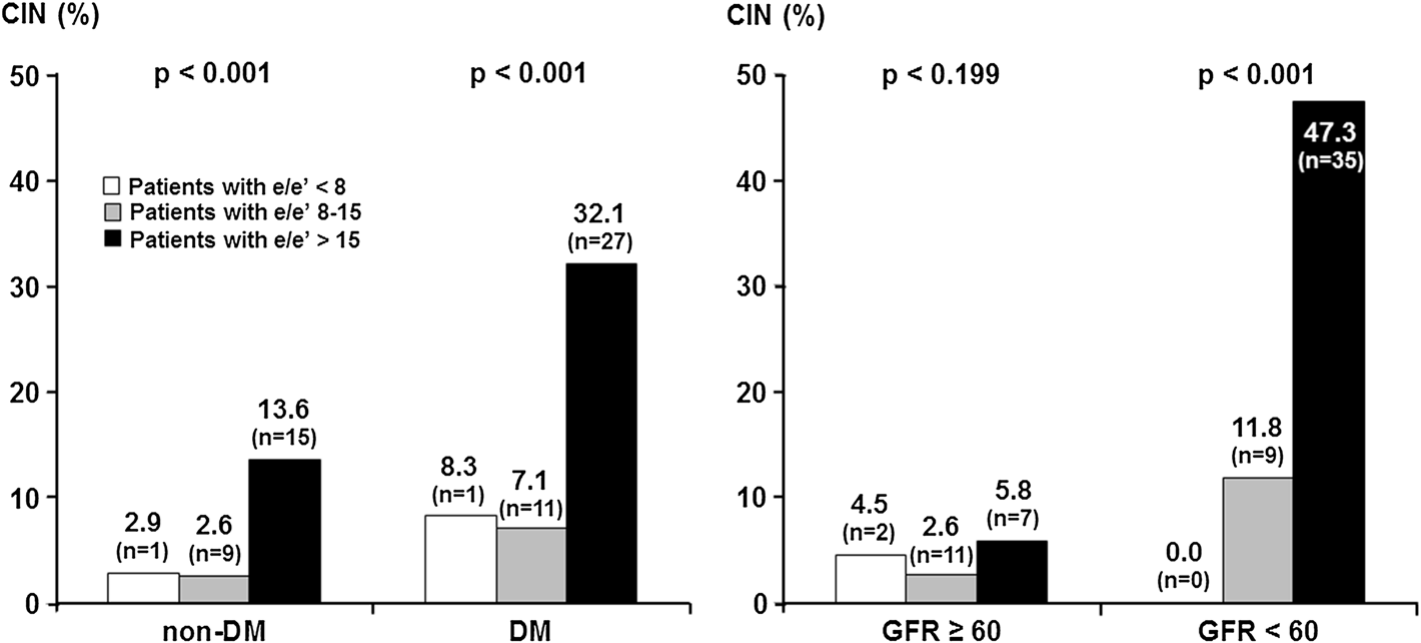
**Relationship between diastolic dysfunction and the incidence of CIN according to the presence of diabetes or renal dysfunction.** CIN developed most frequently in patients in the highest tertile of E/E’ in all subgroups, except for patients with an eGFR ≥ 60 mL/min/1.73m^2^.

In subgroup analysis, CIN occurred most often in patients in the highest tertile of E/E’ irrespective of diabetes status (n = 15, 13.6% in non-DM group, p < 0.001/ n = 27, 32.1% in DM group, p < 0.001). A similar finding was observed in patients with an eGFR < 60 mL/min/1.73m^2^ (n = 35, 47.3% in the highest tertile of E/E’, p < 0.001), although there was no difference in the development of CIN among the three tertiles of E/E’ in patients with an eGFR ≥ 60 mL/min/1.73m^2^ (p = 0.199) (Figure 1).

### Receiver operating characteristic analysis of E/E’ for the development of CIN

**Figure 2:**
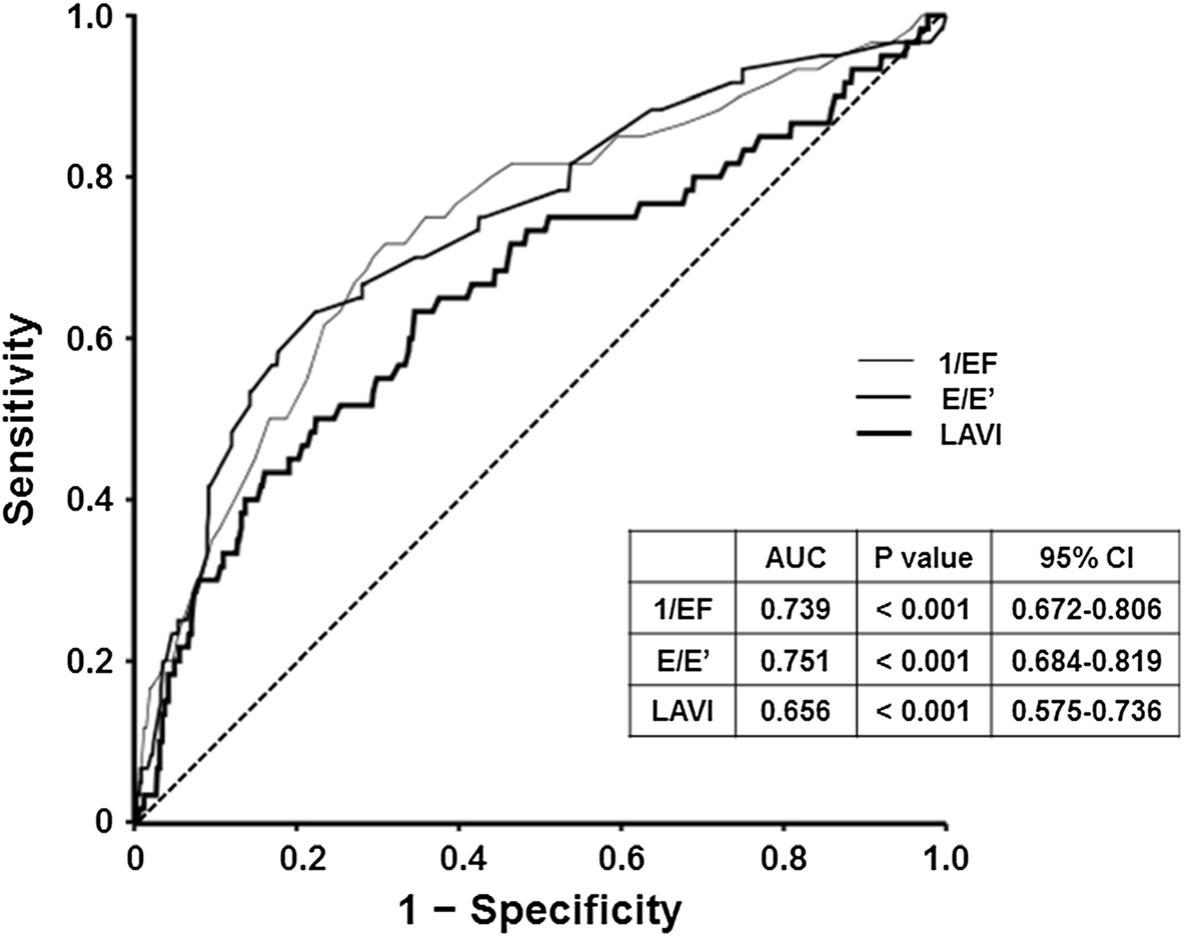
**Receiver operating characteristic curves for the development of CIN according to the echocardiographic variables (EF, E/E’, and LAVI).** The AUCs of EF, E/E, and LAVI were 0.74, 0.75, and 0.66, respectively (p < 0.001).

To estimate the predictive accuracy of the echocardiographic parameters for the development of CIN, ROC analysis was performed. The area under the curve (AUC) for E/E’, EF, and LAVI were 0.75 (95% CI 0.68-0.82, p < 0.001), 0.74 (95% CI 0.67-0.81, p < 0.001), and 0.66 (95% CI 0.58-0.74, p < 0.001), respectively (Figure 2).

When AKIN criteria were applied, estimated AUC of E/E’ for CIN was 0.79 (95% CI 0.73-0.85, p < 0.001) (see Additional file 2).

### Survival analysis in CIN and diastolic dysfunction

During a mean follow-up of 17.9 months, 30 patients died. The most common causes of death were cardiovascular disease (n = 16, 53.5%), followed by infection (n = 12, 40%).

**Figure 3:**
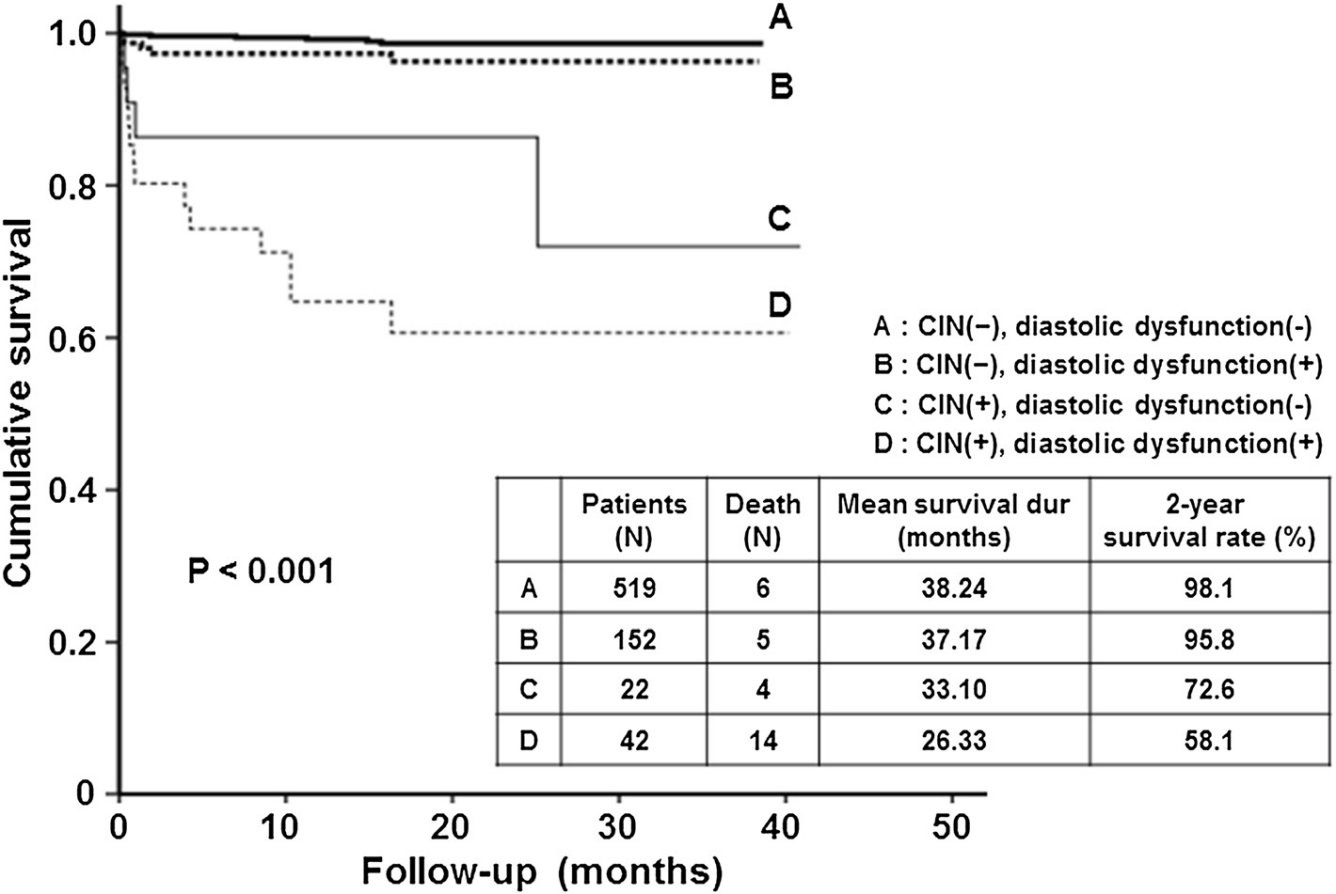
**Kaplan**-**Meier analysis for cumulative hazard of patient mortality**. There was a tendency toward higher mortality in patients with diastolic dysfunction (E/E’ > 15) and/or CIN (p < 0.001).

Kaplan-Meier analysis showed higher mortality rates in patients who developed CIN (n = 18, 28.1% vs. n = 11, 1.6%). In addition, there was a tendency toward higher mortality in those with diastolic dysfunction and CIN (n = 14, 33.3%) (Figure 3). The two-year survival rates were 98.1% in patients without CIN or diastolic dysfunction, 95.8% in patients with diastolic dysfunction but no CIN, 72.6% in subjects with CIN and normal diastolic function, and 58.1% in patients with both CIN and diastolic dysfunction (p < 0.001).

### Comparison of E/E’ with previous risk scoring systems for CIN

We reclassified our patients according to the risk stratification systems suggested by Mehran et al. [14] and Bartholomew et al. [4]. The incidence of CIN was well proportional to both risk scores. Predictive performances for CIN, estimated by AUC on ROC analysis, were about 80% with previous methods and 75% with E/E’ (see Additional file 3).

## Discussion

This study showed that patients with CIN exhibited a lower EF and higher E/E’ and LAVI on echocardiography. In addition, the highest tertile of E/E’ was associated with a significantly increased risk of CIN, beyond the well-known risk factors such as DM and renal failure. This implicates that diastolic dysfunction may be a useful parameter for predicting the development of CIN.

There are both patient-related and patient-unrelated factors in the risk stratification systems for CIN. Patient-related factors include chronic kidney disease (CKD), DM, emergency/ urgent procedure, IABP use, CHF, age > 75 years, hypertension, anemia, a hypotensive episode, and LVEF < 40%. Patient-unrelated factors include using ionic and hyper-osmolar contrast agent, and high volume of CM [9,14]. These factors play a role in the development of CIN primarily by reducing effective renal blood flow, consequently causing hypoxic change and the synthesis of reactive oxygen species. Development of CIN has also been attributed to increased sympathetic tone, renin-angiotensin-aldosterone system (RAAS) activation, the overproduction of many humoral factors such as vasopressin, catecholamines, endothelin, and pro-inflammatory cytokines, and decreased nitric oxide (NO) levels. These hemodynamic and neurohumoral alterations can cause vascular endothelial cell damage and hypertrophic change in the vascular smooth muscle cells, thus further aggravating blood flow disturbances [5,18].

Although CHF is a well-proven risk factor for CIN, only low ejection fraction has previously been studied, among various components of heart failure such as diastolic dysfunction, ventricular hypertrophy, and volume overload [7,8,19]. This has likely been because a decreased effective circulatory volume is considered the primary event in the development of CIN, in patients with CHF [20,21]. However, the statistical significance of EF disappeared in the multivariate logistic regression analysis, whereas E/E’ remained a significant risk factor for CIN (see Additional file 4). ROC analysis also showed a considerable predictive accuracy for E/E’, which was comparable to that of EF (Figure 2). Moreover, most studies demonstrated that EF only affects CIN rates in patients with severe CHF of NYHA class 3–4 or an EF < 30-40%. Given that CIN occurs in higher rates in CHF patients with lower severity, this suggests that the hemodynamic compromise status in milder stages of heart failure cannot be properly represented by EF [8]. Actually, CIN occurred in 18.2% of patients with EF < 40%, and 7.8% of patients with EF ≥ 40% in our study. However, when patients with severe CHF were excluded, E/E’ still was an independent risk factor for CIN (OR 2.593, 95% CI 1.014-6.633, p = 0.041, data not shown). This finding suggests that diastolic dysfunction may be a more reliable parameter to represent the hemodynamic and neurohumoral alterations observed in CHF.

Studies conducted in patients with CHF and preserved LVEF showed a proportional increase in renal failure according to the diastolic dysfunction [22]. The stiffer the LV, the faster the eGFR declined [23]. The main reasons for this include reduced LV functional reserve, resting/ exercise-exacerbated systolic dysfunction, and chronotropic incompetence, which are responsible for the decrease in blood flow. These result in insufficient tissue perfusion and an ischaemic injury to the kidney [24-26]. Initially, LV hypertrophy occurs to compensate for the stiffness of the LV, by increasing stroke volume. But pathologic proliferation, fibrotic deposition, and calcification of the ventricle become evident, and LV compliance eventually decreases [27]. The same vicious cycle in hemodynamics is thought to be the chief mechanism for a higher E/E’ causing CIN. The adverse effect of diastolic dysfunction on patient mortality is partly attributed to these unfavorable geometric changes in the LV (Figure 3).

On the other hand, the neurohumoral changes occurring during the development of CIN further aggravate diastolic dysfunction. Angiotension II and aldosterone promote the growth/ proliferation of both cardiomyocytes and non-myocyte cells like fibroblasts [28]. Pro-inflammatory cytokines such as TNF-α and IL-6 stimulate collagen production by fibroblasts, and bring about a myocardio-depressive effect [29]. We also demonstrated elevated hs-CRP levels in patients in the highest tertile of E/E’ (Table 2). In addition, increased sympathetic tone activates the β-catenin pathway in the cardiomyocytes through the recruitment of Akt (protein kinase B). This up-regulates the production of osteoblastogenic proteins, further aggravating diastolic function [30]. Through these mutual effects on hemodynamic and neurohumoral status, higher E/E’ is thought to cause acute kidney injury when patients with CHF are exposed to the CM.

Furthermore, it should be noted that older age and increased prevalences of hypertension, DM, and 3-VD were present in subjects with diastolic dysfunction (Table 2). These comorbidities are well-proven risk factors, and may potentiate the relationship between diastolic dysfunction and CIN. In DM, advanced glycation endproduct (AGE) mediates the crosslinking of collagen fibres in the myocardium [31]. It also causes an inflammation in the renal vascular system after binding to the receptor of AGE (RAGE), through activation of NADPH oxidase, MAP kinase, and the NF-κB pathway [32,33]. Because insulin and c-peptide are known to evoke the overexpression of inducible endothelial NO synthase, impaired pancreatic secretion is also expected to increase E/E’ and exacerbate renal hemodynamics [34,35]. Actually, several studies demonstrated an improved ejection fraction/ diastolic function after correcting for these metabolic abnormalities in diabetic CKD patients with a kidney-pancreas co-transplantation [36,37]. Underlying hypertension and CKD also strengthen the linkage between diastolic dysfunction and CIN through the effects of uremic toxin [38,39], pressure overload, RAAS activation, and markedly elevated expression of humoral mediators such as catecholamines, endothelin, and parathyroid hormone [40].

There are several shortcomings to our study. Because this was a retrospective study, limited data were available, which may have affected the conclusion by type II error. Second, as estimating LVEDP during the PTCA was not a routine practice in our institute, LVEDP and their relationships with E/E’ and CIN could not be presented. However, in other small cohort of 55 patients who measured LVEDP during the PTCA in our hospital, E/E’ levels were well correlated with LVEDP (r = 0.78, p < 0.001). The ROC analysis demonstrated that the predictive accuracy of E/E’ for LVEDP > 15mmHg was 0.88 (p < 0.001, 95% CI 0.794-0.981). E/E’ > 15 had 81% sensitivity and 86% specificity for LVEDP > 15 mmHg (see Additional file 5). These results correspond closely with previous studies by Sohn et al. and Ommen et al. [11-13]. Moreover, E/E’ can be measured non-invasively prior to PTCA, and warns the physicians to estimate LVEDP during procedure and to monitor patients’ renal function carefully after procedure, in advance, when levels are elevated. Third, as only patients who were suspected to have CAOD and had undergone an echocardiography were included, selection bias may have existed. Higher incidence rate of CIN in our study group compared to that in previous studies conducted in the general population supports this [1]. However, considering that interventional procedures using intravascular CM are most closely related with the development of CIN, and most patients requiring such procedures have underlying cardiac disease and DM, the results of present study can be applied to the high-risk patients planning for the angioplasty. The uncontrolled use of medications with vasomotor action or that modulate the neurohumoral axis is another limitation. However, former clinical trials using RAAS blockade, statins, calcium channel blockers, beta-blockers, and diuretics failed to derive a common consensus as to their effects on CIN [41-43]. Lastly, as it was a single-center study, further large-scale, randomized controlled multicenter trials are needed to confirm and assess the clinical applicability of our findings.

## Conclusions

In conclusion, we demonstrated that, among echocardiographic parameters, E/E’ can be a useful predictor for the development of CIN. To our knowledge, this is the first study suggesting that assessment of diastolic dysfunction should play a role in the risk stratification for CIN.

## Abbreviations

AGE: Advanced glycation endproduct; AKIN: Acute Kidney Injury Network; ANP: Atrial natriuretic peptide; ASE: American Society of Echocardiography; AUC: Area under the curve; BMI: Body mass index; CAOD: Coronary artery occlusive disease; CHF: Congestive heart failure; CI: Confidence interval; CIN: Contrast-induced nephropathy; CKD: Chronic kidney disease; CM: Contrast media; DT: Deceleration time; EF: Ejection fraction; eGFR: Estimated glomerular filtration rate; Hs-CRP: High-sensitivity C-reactive protein; IABP: Intra-aortic balloon pump; IVS: Interventricular septal thickness; LAVI: Left atrial volume index; LVDd: Left ventricular end-diastolic dimension; LVDs: Left ventricular end-systolic dimension; LVEDP: Left ventricular end-diastolic pressure; LVM: Left ventricular mass; LVMI: Left ventricular mass index; MDRD: Modification of Diet in Renal Disease; NO: Nitric oxide; NYHA: New York Heart Association; OR: Odds ratio; PTCA: Percutaneous transluminal coronary angioplasty; PWT: Posterior wall thickness; RAAS: Renin-angiotensin-aldosterone system; ROC: Receiver operating characteristic; 3-VD: Three-vessel coronary artery disease.

## Competing interests

The authors declare that they have no competing interests.

## Authors’ contributions

HMK designed the study, acquired the data, performed the statistical analysis, interpreted the analytical results, and wrote the manuscript; SHH, THY and SWK edited and made critical revision to the manuscript; HJO assisted in the study design and helped draft the manuscript; BSK participated in the data analysis and interpretation; FMD, KIK, CHK, and MJL assisted in the study design and contributed to the data acquisition; KHC organized the data collection and edited the manuscript. All authors approved the final version of the manuscript.

## Pre-publication history

The pre-publication history for this paper can be accessed here:

http://www.biomedcentral.com/1471-2369/14/146/prepub

## Supplementary Material

Additional file 1 Univariate logistic regression analysis for contrast-induced nephropathy, which was defined according to the AKIN criteria. Click here for file

Additional file 2 **Receiver operating characteristic curves for CIN, which was defined according to the AKIN criteria.** The AUCs of EF, E/E, and LAVI were 0.70, 0.79, and 0.69, respectively (p < 0.001). Click here for file

Additional file 3 **Predictive performances for CIN using various risk scoring systems.** The incidence of CIN was well proportional to both (a) Mehran’s (renal failure was scored according to the eGFR: white bar, or the Cr levels: black bar) and (b) Bartholomew’s risk scores. (c) E/E’ showed a considerable predictive power for the development of CIN, which was comparable to other risk stratification methods. The AUCs of E/E’ , Mehran’s score, and Bartholomew’s score were 0.75, 0.80, and 0.82, respectively (p < 0.001). Click here for file

Additional file 4 **Multivariate logistic regression analysis for contrast-induced nephropathy (detailed descriptions of Table** 4). Click here for file

Additional file 5 **Correlation between E/E’ and LVEDP.** (a) E/E’ showed a significant positive relationship with LVEDP on correlation analysis. (b) Across increasing E/E’ tertiles, LVEDP levels were incrementally higher. (c) ROC analysis revealed that the predictive accuracy of E/E’ for LVEDP > 15 mmHg was 0.88 (p < 0.001, 95% CI 0.794-0.981). Click here for file
