# Supplementary material for: Diastolic dysfunction is associated with an increased risk of contrast-induced nephropathy: a retrospective cohort study
Source: BMC Nephrol. 2013 Jul 13;14:146. doi: 10.1186/1471-2369-14-146 (PMC3717078; doi:10.1186/1471-2369-14-146)
Supplement: Additional file 1 — Univariate logistic regression analysis for contrast-induced nephropathy, which was defined according to the AKIN criteria. [file 1471-2369-14-146-S1.docx]

## **Table 1 - Univariate logistic regression analysis for contrast-induced nephropathy, which was defined according to the AKIN criteria.**

| **Variables** | **OR** | **95% CI** | ***p*** |
| --- | --- | --- | --- |
| **Age > 75 years (vs. ≤ 75 years)** | 3.399 | 1.945-5.942 | <0.001 |
| **BMI (kg/m^2^)** | 0.834 | 0.761-0.915 | <0.001 |
| **Hypertension** | 3.715 | 1.733-7.962 | 0.001 |
| **Diabetes mellitus** | 3.533 | 2.029-6.151 | <0.001 |
| **Emergency/ urgent procedure**  **(vs. elective procedure)** | 2.015 | 1.137-3.572 | 0.016 |
| **3-vessel disease (vs. < 3-vessel involvement)** | 4.817 | 2.717-8.539 | <0.001 |
| **IABP use (vs. non-IABP use)** | 19.429 | 8.769-43.046 | <0.001 |
| **Volume of CM per weight (mL/kg)** | 1.269 | 1.031-1.561 | 0.024 |
| **eGFR < 60 mL/min/1.73m^2^**  **(vs. eGFR ≥ 60 mL/min/1.73m^2^)** | 18.408 | 9.602-35.288 | <0.001 |
| **Hemoglobin (g/dL)** | 0.555 | 0.474-0.651 | <0.001 |
| **Albumin (g/dL)** | 0.181 | 0.111-0.296 | <0.001 |
| **hs-CRP (mg/L)** | 1.015 | 1.009-1.022 | <0.001 |
| **E/E'** | 1.166 | 1.118-1.217 | <0.001 |
| **E/E' > 15 (vs. E/E’ ≤ 15)** | 7.596 | 4.233-13.631 | <0.001 |
| **EF ≤ 40% (vs. EF > 40%)** | 2.845 | 1.483-5.458 | 0.002 |
| **LAVI > 35 mL/m^2^ (vs. LAVI ≤ 35 mL/m^2^)** | 4.788 | 2.703-8.482 | <0.001 |

Data are presented as odds ratios (OR) and 95% confidence intervals (CI).

*Abbreviations* BMI, body mass index; IABP, intra-aortic balloon pump; CM, contrast media; eGFR, estimated glomerular filtration rate; hs-CRP, high sensitivity C-reactive protein; EF, ejection fraction; LAVI, left atrial volume index

## **Table 2. Odds ratios and 95% confidence intervals for contrast-induced nephropathy according to the echocardiographic parameter E/E’ (Multivariate logistic regression analysis).**

|  | **E/E’** | | |
| --- | --- | --- | --- |
|  | **OR** | **95% CI** | ***p*** |
| **^a^Model 1** | 1.102 | 1.034-1.173 | 0.003 |
| **^b^Model 2** | 3.190 | 1.336-7.621 | 0.009 |
| **^c^Model 3** | 3.214 | 1.322-7.811 | 0.010 |
| **^d^Model 4** | 2.456 | 1.046-6.217 | 0.044 |

^a^ Model 1 (odds ratio per 1 increase in E/E’) : adjusted for age (> 75 years vs. ≤ 75 years), BMI, hypertension, diabetes, emergent/ urgent procedure, 3-vessel disease, IABP use, volume of CM per weight, eGFR (< 60 mL/min/1.73m^2^ vs. eGFR ≥ 60 mL/min/1.73m^2^), hemoglobin, albumin, and hs-CRP

^b^ Model 2 (odds ratio for E/E’ > 15 vs. E/E’ ≤ 15) : adjusted for age (> 75 years vs. ≤ 75 years), BMI, hypertension, diabetes, emergent/ urgent procedure, 3-vessel disease, IABP use, volume of CM per weight, eGFR (< 60 mL/min/1.73m^2^ vs. eGFR ≥ 60 mL/min/1.73m^2^), hemoglobin, albumin, and hs-CRP

^c^ Model 3 (odds ratio for E/E’ > 15 vs. E/E’ ≤ 15) : adjusted for Model 2 plus ejection fraction (≤ 40% vs. > 40%)

^d^ Model 4 (odds ratio for E/E’ > 15 vs. E/E’ ≤ 15) : adjusted for Model 3 plus left atrial volume index (> 35 vs. ≤ 35)

*Abbreviations* BMI, body mass index; IABP, intra-aortic balloon pump; CM, contrast media; eGFR, estimated glomerular filtration rate; hs-CRP, high sensitivity C-reactive protein
