# Supplementary figures and images for: Diastolic dysfunction is associated with an increased risk of contrast-induced nephropathy: a retrospective cohort study
Source: BMC Nephrol. 2013 Jul 13;14:146. doi: 10.1186/1471-2369-14-146 (PMC3717078; doi:10.1186/1471-2369-14-146)

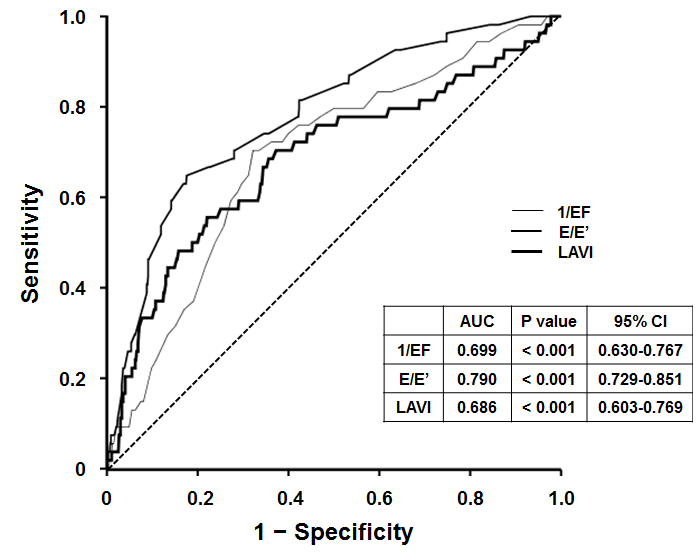

Supplement: Additional file 2 — Receiver operating characteristic curves for CIN, which was defined according to the AKIN criteria. The AUCs of EF, E/E, and LAVI were 0.70, 0.79, and 0.69, respectively (p < 0.001). [file 1471-2369-14-146-S2.tiff]

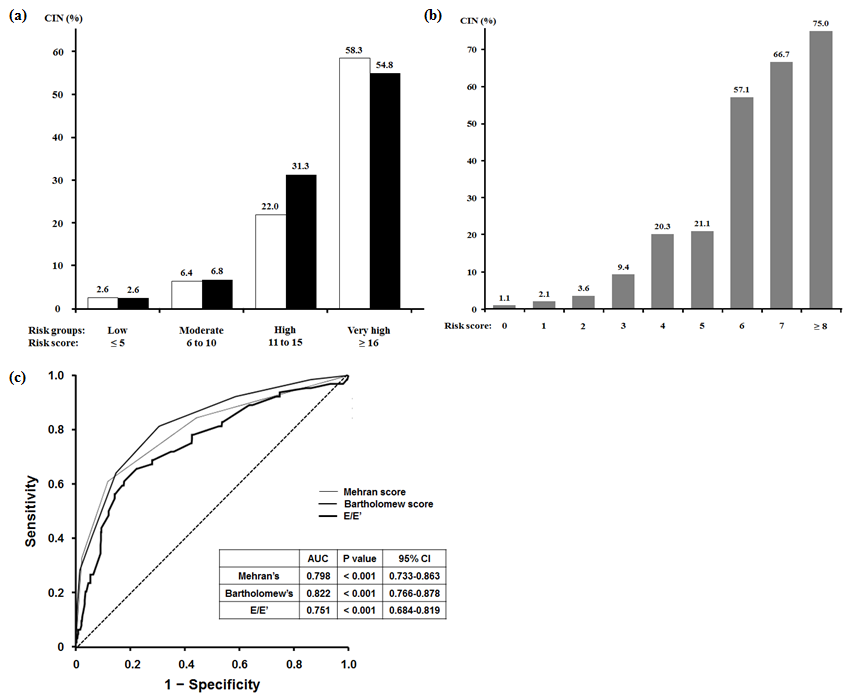

Supplement: Additional file 3 — Predictive performances for CIN using various risk scoring systems. The incidence of CIN was well proportional to both (a) Mehran’s (renal failure was scored according to the eGFR: white bar, or the Cr levels: black bar) and (b) Bartholomew’s risk scores. (c) E/E’ showed a considerable predictive power for the development of CIN, which was comparable to other risk stratification methods. The AUCs of E/E’ , Mehran’s score, and Bartholomew’s score were 0.75, 0.80, and 0.82, respectively (p < 0.001). [file 1471-2369-14-146-S3.tiff]

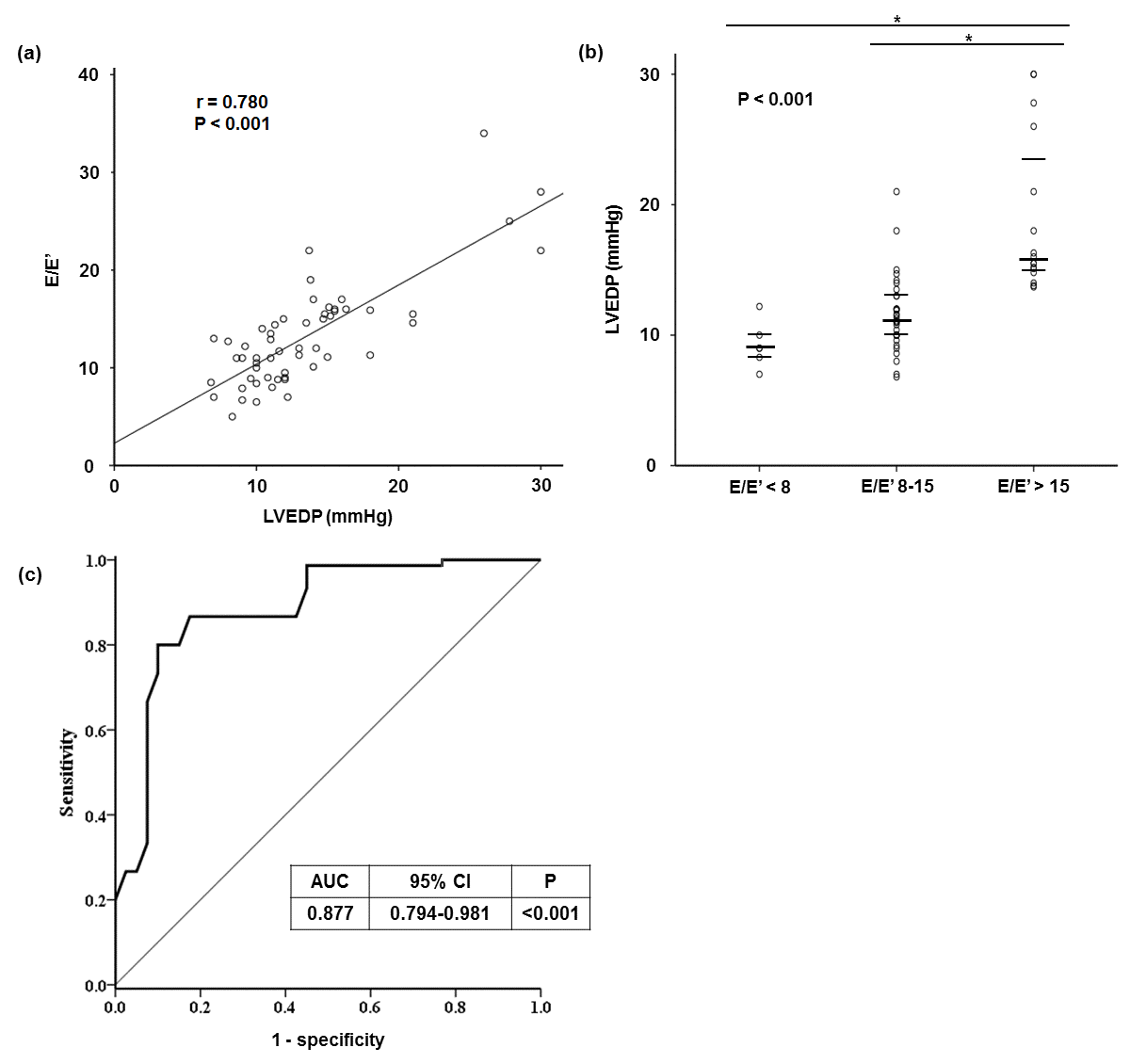

Supplement: Additional file 5 — Correlation between E/E’ and LVEDP. (a) E/E’ showed a significant positive relationship with LVEDP on correlation analysis. (b) Across increasing E/E’ tertiles, LVEDP levels were incrementally higher. (c) ROC analysis revealed that the predictive accuracy of E/E’ for LVEDP > 15 mmHg was 0.88 (p < 0.001, 95% CI 0.794-0.981). [file 1471-2369-14-146-S5.tiff]
