# Supplementary material for: Diastolic dysfunction is associated with an increased risk of contrast-induced nephropathy: a retrospective cohort study
Source: BMC Nephrol. 2013 Jul 13;14:146. doi: 10.1186/1471-2369-14-146 (PMC3717078; doi:10.1186/1471-2369-14-146)
Supplement: Additional file 4 — Multivariate logistic regression analysis for contrast-induced nephropathy (detailed descriptions of Table 4). [file 1471-2369-14-146-S4.docx]

## **Table 3 - Multivariate logistic regression analysis for contrast-induced nephropathy (detailed descriptions of Table 4).**

## **Model 1.**

| **Variables** | **OR** | **95% CI** | ***p*** |
| --- | --- | --- | --- |
| **Age > 75 years (vs. ≤ 75 years)** | 0.868 | 0.382-1.975 | 0.736 |
| **BMI (kg/m^2^)** | 0.840 | 0.730-0.967 | 0.015 |
| **Hypertension** | 1.593 | 0.591-4.291 | 0.357 |
| **Diabetes mellitus** | 2.060 | 1.035-5.747 | 0.042 |
| **Emergency/ urgent procedure**  **(vs. elective procedure)** | 2.525 | 0.972-6.559 | 0.057 |
| **3-vessel disease (vs. < 3-vessel involvement)** | 2.346 | 1.064-5.174 | 0.035 |
| **IABP use (vs. non-IABP use)** | 4.920 | 1.398-17.315 | 0.013 |
| **Volume of CM per weight (mL/kg)** | 1.002 | 0.756-1.328 | 0.986 |
| **eGFR < 60 mL/min/1.73m^2^**  **(vs. eGFR ≥ 60 mL/min/1.73m^2^)** | 3.371 | 1.415-8.034 | 0.006 |
| **Hemoglobin (g/dL)** | 0.927 | 0.705-1.220 | 0.589 |
| **Albumin (g/dL)** | 0.723 | 0.291-1.792 | 0.483 |
| **hs-CRP (mg/L)** | 1.012 | 1.003-1.022 | 0.014 |
| **E/E'** | 1.091 | 1.026-1.159 | 0.005 |

Data are presented as odds ratios (OR) and 95% confidence intervals (CI).

*Abbreviations* BMI, body mass index; IABP, intra-aortic balloon pump; CM, contrast media; eGFR, estimated glomerular filtration rate; hs-CRP, high sensitivity C-reactive protein

## **Model 2.**

| **Variables** | **OR** | **95% CI** | ***p*** |
| --- | --- | --- | --- |
| **Age > 75 years (vs. ≤ 75 years)** | 0.848 | 0.378-1.900 | 0.688 |
| **BMI (kg/m^2^)** | 0.843 | 0.731-0.972 | 0.019 |
| **Hypertension** | 1.696 | 0.625-4.602 | 0.300 |
| **Diabetes mellitus** | 2.213 | 1.047-4.952 | 0.038 |
| **Emergency/ urgent procedure**  **(vs. elective procedure)** | 2.369 | 0.910-6.166 | 0.077 |
| **3-vessel disease (vs. < 3-vessel involvement)** | 2.234 | 1.007-4.958 | 0.048 |
| **IABP use (vs. non-IABP use)** | 4.678 | 1.295-16.905 | 0.019 |
| **Volume of CM per weight (mL/kg)** | 0.980 | 0.740-1.297 | 0.887 |
| **eGFR < 60 mL/min/1.73m^2^**  **(vs. eGFR ≥ 60 mL/min/1.73m^2^)** | 3.696 | 1.582-8.637 | 0.003 |
| **Hemoglobin (g/dL)** | 0.923 | 0.702-1.213 | 0.566 |
| **Albumin (g/dL)** | 0.740 | 0.301-1.821 | 0.513 |
| **hs-CRP (mg/L)** | 1.013 | 1.003-1.022 | 0.010 |
| **E/E' > 15 (vs. E/E’ ≤ 15)** | 3.435 | 1.522-7.755 | 0.003 |

Data are presented as odds ratios (OR) and 95% confidence intervals (CI).

*Abbreviations* BMI, body mass index; IABP, intra-aortic balloon pump; CM, contrast media; eGFR, estimated glomerular filtration rate; hs-CRP, high sensitivity C-reactive protein

## **Model 3.**

| **Variables** | **OR** | **95% CI** | ***p*** |
| --- | --- | --- | --- |
| **Age > 75 years (vs. ≤ 75 years)** | 0.860 | 0.382-1.939 | 0.717 |
| **BMI (kg/m^2^)** | 0.846 | 0.733-0.978 | 0.023 |
| **Hypertension** | 1.697 | 0.625-4.606 | 0.299 |
| **Diabetes mellitus** | 2.185 | 1.026-4.906 | 0.041 |
| **Emergency/ urgent procedure**  **(vs. elective procedure)** | 2.280 | 0.847-6.132 | 0.103 |
| **3-vessel disease (vs. < 3-vessel involvement)** | 2.184 | 0.971-4.910 | 0.059 |
| **IABP use (vs. non-IABP use)** | 4.329 | 1.092-17.171 | 0.037 |
| **Volume of CM per weight (mL/kg)** | 0.986 | 0.743-1.308 | 0.921 |
| **eGFR < 60 mL/min/1.73m^2^**  **(vs. eGFR ≥ 60 mL/min/1.73m^2^)** | 3.658 | 1.559-8.587 | 0.003 |
| **Hemoglobin (g/dL)** | 0.923 | 0.702-1.214 | 0.567 |
| **Albumin (g/dL)** | 0.726 | 0.293-1.800 | 0.490 |
| **hs-CRP (mg/L)** | 1.012 | 1.002-1.022 | 0.017 |
| **E/E' > 15 (vs. E/E’ ≤ 15)** | 3.344 | 1.456-7.682 | 0.004 |
| **EF ≤ 40% (vs. EF > 40%)** | 1.177 | 0.417-3.316 | 0.158 |

Data are presented as odds ratios (OR) and 95% confidence intervals (CI).

*Abbreviations* BMI, body mass index; IABP, intra-aortic balloon pump; CM, contrast media; eGFR, estimated glomerular filtration rate; hs-CRP, high sensitivity C-reactive protein; EF, ejection fraction

## **Model 4.**

| **Variables** | **OR** | **95% CI** | ***p*** |
| --- | --- | --- | --- |
| **Age > 75 years (vs. ≤ 75 years)** | 0.875 | 0.383-1.997 | 0.750 |
| **BMI (kg/m^2^)** | 0.867 | 0.749-1.003 | 0.055 |
| **Hypertension** | 1.705 | 0.619-4.699 | 0.302 |
| **Diabetes mellitus** | 2.379 | 1.052-5.379 | 0.037 |
| **Emergency/ urgent procedure**  **(vs. elective procedure)** | 2.538 | 0.934-6.897 | 0.068 |
| **3-vessel disease (vs. < 3-vessel involvement)** | 2.012 | 0.880-4.599 | 0.097 |
| **IABP use (vs. non-IABP use)** | 5.087 | 1.153-20.444 | 0.032 |
| **Volume of CM per weight (mL/kg)** | 1.003 | 0.753-1.337 | 0.982 |
| **eGFR < 60 mL/min/1.73m^2^**  **(vs. eGFR ≥ 60 mL/min/1.73m^2^)** | 3.463 | 1.448-8.284 | 0.005 |
| **Hemoglobin (g/dL)** | 0.929 | 0.706-1.224 | 0.601 |
| **Albumin (g/dL)** | 0.746 | 0.291-1.909 | 0.541 |
| **hs-CRP (mg/L)** | 1.012 | 1.002-1.023 | 0.021 |
| **E/E' > 15 (vs. E/E’ ≤ 15)** | 2.579 | 1.082-5.964 | 0.035 |
| **EF ≤ 40% (vs. EF > 40%)** | 1.167 | 0.405-3.359 | 0.354 |
| **LAVI > 35 mL/m^2^ (vs. LAVI ≤ 35 mL/m^2^)** | 2.117 | 0.890-5.036 | 0.090 |

Data are presented as odds ratios (OR) and 95% confidence intervals (CI).

*Abbreviations* BMI, body mass index; IABP, intra-aortic balloon pump; CM, contrast media; eGFR, estimated glomerular filtration rate; hs-CRP, high sensitivity C-reactive protein; EF, ejection fraction; LAVI, left atrial volume index
